# Comparative analyses of functional antibody-mediated inhibition with anti-circumsporozoite monoclonal antibodies against transgenic *Plasmodium berghei*

**DOI:** 10.1186/s12936-023-04765-2

**Published:** 2023-11-07

**Authors:** Justin Nicholas, Surendra Kumar Kolli, Pradeep Annamalai Subramani, Sai Lata De, Madison M. Ogbondah, Samantha J. Barnes, Francis Babila Ntumngia, John H. Adams

**Affiliations:** 1 Center for Global Health and Interdisciplinary Research, College of Public Health, University of South Florida, 3720 Spectrum Blvd, Tampa, FL 33612 USA; 2 Department of Molecular Medicine, Morsani College of Medicine, University of South Florida, Tampa, FL 33612 USA; 3 Present Address: Department of Infectious Disease & Immunology, College of Veterinary Medicine, University of Florida, Gainesville, FL 32611 USA

**Keywords:** Transgenic, *Plasmodium berghei*, In vitro assay, Correlation, Challenge, Monoclonal antibody, Circumsporozoite protein

## Abstract

**Background:**

Acquired functional inhibitory antibodies are one of several humoral immune mechanisms used to neutralize foreign pathogens. In vitro bioassays are useful tools for quantifying antibody-mediated inhibition and evaluating anti-parasite immune antibodies. However, a gap remains in understanding of how antibody-mediated inhibition in vitro translates to inhibition in vivo. In this study, two well-characterized transgenic *Plasmodium berghei* parasite lines, *Pb*mCh-luc and *Pb*-*Pf*CSP(r), and murine monoclonal antibodies (mAbs) specific to *P. berghei* and *Plasmodium falciparum* circumsporozoite protein (CSP), 3D11 and 2A10, respectively, were used to evaluate antibody-mediated inhibition of parasite development in both in vitro and in vivo functional assays.

**Methods:**

IC_50_ values of mAbs were determined using an established inhibition of liver-stage development assay (ILSDA). For the in vivo inhibition assay, mice were passively immunized by transfer of the mAbs and subsequently challenged with 5.0 × 10^3^ sporozoites via tail vein injection. The infection burden in both assays was quantified by luminescence and qRT-PCR of *P. berghei* 18S rRNA normalized to host GAPDH.

**Results:**

The IC_50_ values quantified by relative luminescence of mAbs 3D11 and 2A10 were 0.396 µg/ml and 0.093 µg/ml, respectively, against transgenic lines in vitro. Using the highest (> 90%) inhibitory antibody concentrations in a passive transfer, an IC_50_ of 233.8 µg/ml and 181.5 µg/ml for mAbs 3D11 and 2A10, respectively, was observed in vivo. At 25 µg (250 µg/ml), the 2A10 antibody significantly inhibited liver burden in mice compared to control. Additionally, qRT-PCR of *P. berghei* 18S rRNA served as a secondary validation of liver burden quantification.

**Conclusions:**

Results from both experimental models, ILSDA and in vivo challenge, demonstrated that increased concentrations of the homologous anti-CSP repeat mAbs increased parasite inhibition. However, differences in antibody IC_50_ values between parasite lines did not allow a direct correlation between the inhibition of sporozoite invasion in vitro by ILSDA and the inhibition of mouse liver stage burden. Further studies are needed to establish the conditions for confident predictions for the in vitro ILSDA to be a predictor of in vivo outcomes using this model system.

**Supplementary Information:**

The online version contains supplementary material available at 10.1186/s12936-023-04765-2.

## Background

In 2021, malaria infections accounted for an estimated 247 million cases and 619,000 deaths, with the majority of the burden afflicting the African region [1]. This global disease is transmitted to humans by the bite of an infected *Anopheles* spp*.* mosquito during a blood meal, releasing sporozoites into the dermis and initiating an infection [2]. These sporozoites then migrate to blood vessels by active gliding motility [3–5] to reach the liver sinusoid. The sporozoites traverse through hepatic tissue before invading a hepatocyte to begin asexual schizogony [6, 7]. The rupture of hepatocytes releases merozoites which invade red blood cells initiating the blood-stage infection. Although the blood stage is associated with morbidity and mortality from malaria, the sporozoites and liver-stage (LS) forms of the pre-erythrocytic (PE) stage represent ideal therapeutic targets for disease protection [8, 9]. RTS,S/AS01 took advantage of this stage’s vulnerability, leading to the first malaria vaccine. RTS,S targets *Plasmodium falciparum* CSP and achieves 70.6% efficacy against severe malaria when given seasonally with chemopreventative drugs [10–12].

CSP is a multifunctional parasite protein essential for sporozoite development and hepatic infections [13–15]. The N-terminal region of CSP consists of a junctional region and conserved pentamer ‘KLKQP’ termed region 1 [13]. CSP’s C-terminal domain comprises of a known cell-adhesive motif called type I thrombospondin repeat (TSR), termed region II, and a GPI anchor [13]. In between the N- and C-terminal domains is a central immunodominant repeat region comprising of 4 to 8 tandem amino acid repeats [16, 17]. Earlier studies have shown that these repeats are species-specific, and antibodies targeting the repeat region immobilize sporozoites and block hepatocyte invasion [16, 18, 19]. Later studies corroborated these findings with sporozoite challenge studies in mice and humans, correlating this protection with anti-CSP sera [20–24]. These early challenge studies provided a rationale for CSP as a PE stage vaccine candidate [25].

In vitro*,* liver-stage functional assays are essential for the preclinical evaluation of PE-stage vaccine candidates against malaria parasites [26–32]. These assays initially characterize antibodies that can interrupt critical sporozoite invasive phenotypes, such as gliding, cell traversal, and liver-stage development [32–38]. Similarly, rodent models have been used for decades to evaluate the safety, immunogenicity, and efficacy of vaccine candidates and elucidate parasite biology [39–44]. These models are advantageous when assessing correlates of humoral protection against the PE stage in vivo [45]. As antibody-mediated inhibition in vitro reflects the potential for inhibition in vivo [46]; it is important to understand the predictive power of in vitro bioassays to in vivo outcomes.

Here, the correlation of antibody-mediated inhibition between two well-characterized anti-CSP mAbs against the central repeat regions, 3D11 (*Plasmodium berghei*) and 2A10 (*P. falciparum*), was analysed in vitro and in vivo*,* using transgenic *P. berghei* sporozoites [47–50]. These antibodies were selected based on data from previous studies which demonstrated the functional inhibition of these mAbs against critical PE stage phenotypes [32, 51]. Functional inhibition was characterized by performing sporozoite live gliding motility and inhibition of liver-stage development assays (ILSDA) followed by a homologous challenge in mice. This study provides valuable insight into the use of in vitro bioassays to predict anti-CSP antibody function in vivo. However, a direct correlation between the in vitro assays and challenge model could not be established suggesting the need for further studies to precisely identify in vitro assay factors that are reliable predictors of in vivo outcomes.

## Methods

### Ethics statement

4 to 6 weeks female BALB/c mice (Envigo) were maintained under pathogen-free conditions following the guidelines set by the Institutional Animal Care and Use Committee (IACUC) protocol IS000010943R approved by the University of South Florida Ethics Committee.

### Transgenic *Plasmodium berghei* parasite lines

Two *P. berghei* transgenic parasite lines were used. The first, a *Pb*ANKA-*Pf*CSP(r) *Pb*CSP termed *Pb-Pf*CSP(r) (2257cl2), expressing *Pf*CSP (PF3D7_0304600) in place of endogenous *Pb*CSP (PBANKA_0403200) and a GFP-luciferase fusion reporter (GFP::Luc_Pbeef1α_) introduced at a neutral 230p locus. A second transgenic line *Pb*ANKA-mCherry_hsp70_ + Luc_eef1α_ termed *Pb*mCh-luc (1868cl1), that express *mCherry* and *luciferase* reporters under *hsp70* and *eef1α* promoters, respectively at neutral 230p locus. Transgenic lines were obtained from the repository www.pberghei.eu labelled RMgm-4110 (*Pb-Pf*CSP(r)) and RMgm-1320 (*Pb*mCh-luc). Transgenic line *Pb*mCh-luc was chosen as a pseudo-wildtype (‘WT’) instead of *P. berghei-*ANKA to facilitate in vitro and in vivo quantification. Both lines were previously described [52–54].

### Sporozoite propagation

6 to 8 weeks female BALB/c mice (Envigo) were injected intraperitonially with 100 to 150 µl of cryopreserved parasitized blood of either *Pb*-*Pf*CSP(r) or *Pb*mCh-luc. At 0.3% to 0.5% gametocytaemia, anaesthetized mice were fed to 3- to-5-day-old female *Anopheles stephensi* mosquitoes. The blood-fed mosquitoes were maintained at 21 °C in 70–80% relative humidity. The infectivity status of the blood-fed mosquitoes was assessed on days 10–12 for oocyst formation in the midgut and on days 18–21 for sporozoites in the salivary glands. The mosquitoes were anaesthetized by cold treatment and surface sterilized before salivary gland dissection, as previously described [55]. Sporozoites were prepared aseptically and quantified for experiments.

### Passive immunizations and challenge

Groups of mice (n = 4) were administered intraperitoneally (IP) with 25, 12.5, or 2.5 µg of 3D11 and 2A10 in 100 µL of PBS 30 min before challenge. These concentrations were equivalent to 250, 125, and 25 µg/ml concentrations in vitro. Control mice received PBS. Following mAb injection, mice were challenged with 100 µl of 5.0 × 10^3^ sporozoites resuspended in RPMI (Corning) via tail IV injections. Bioluminescence was measured at 44 h post infection (hpi) imaged using in vivo imaging system (IVIS) (PerkinElmer) as described below. Liver samples were collected for RNA isolation and snap-frozen in liquid nitrogen.

### Visualization and quantification of luciferase activity in mice challenged with transgenic *P. berghei*

Liver-stage luciferase activity was visualized using an intensified-charge-coupled device (I-CCD) video camera of the IVIS. Mice were anaesthetized using isoflurane anesthesia system (XGI-8, Xenogen). Following this, mice were injected intraperitoneally (IP) with d-luciferin (100 mg/kg of body weight) (Xenolight, Perkin Elmer,) in PBS. Measurements were performed within 5 min of injection. Bioluminescence imaging was acquired on auto-exposure. Imaging data were analysed using the living image 3.0 (Xenogen) software. Quantitative luminescence reads with equal region of interest (ROI) across all mice abdomens were measured. Total flux (photons/second [p/s]) values were calculated from the ROI and used to determine relative infectivity.

### Hepatoma culture

Hepatoma cells (HC-04) were maintained in Minimum Essential Media (Gibco) and Ham’s F12 nutrient mix (Gibco), supplemented with 30 mM HEPES (Gibco), 10% heat-inactivated fetal bovine serum (Sigma-Aldrich), 2 mM l-Glutamine (Gibco) and 40 µg/ml Gentamicin (Sigma-Aldrich) at 37 °C (5% CO_2_). At ~ 90% confluency, cells were split 1:6 into a pre-coated collagen flask (Corning™) every 3–4 days. The cell line was authenticated via American Type Culture Collection Human STR Profiling Service (ATCC) and tested negative for mycoplasma contamination (Fisher Scientific).

### Live gliding motility assay

The live gliding motility assay was performed as previously described with minor changes [56]. Briefly, 384-well glass-bottom plates (Greiner Bio-One) were placed at 37 °C in a CO_2_ incubator. The mAbs were diluted (500, 5 and 0.5 μg/ml) in 6% BSA in RPMI-1640 medium (Corning) in 80 μl and incubated at 37 °C in a CO_2_ incubator. After salivary gland dissection and sporozoite preparation, 80 μl of sporozoites (150 sporozoites/µl) was added to each mAb dilution and control and 40 μl was dispensed to each well in triplicates. Plates were centrifuged at 200×*g* for 3 min and imaged using Cell Insight CX7 high-content imaging system (Thermo Fisher Scientific) in brightfield. Wells were imaged at 20X at the rate of one frame/second for 60 s. Acquired images were exported for analysis and sporozoites were manually quantified as gliding and non-gliding. Gliding was defined as attached movement, and percent gliding was calculated by dividing total gliding sporozoites by total sporozoites.

### Inhibition of liver-stage development assay (ILSDA)

ILSDA was carried out as previously described with minor changes [32]. HC-04 cells were seeded at a density of 8000 cells per well in a 384 well plate (Greiner Bio-One), 24 h before infection with sporozoites. After the aseptic preparation of sporozoites, 75 sporozoites/µl were incubated at room temperature with mAbs at a final concentration of 250, 125, 25, 2.5, 0.25, and 0.025 μg/ml for 20 min. 2A10 and 3D11 mAbs were an isotype control for PbmCh-luc and Pb-PfCSP, respectively. No antibody-treated sporozoites served as controls and were used for data normalization. Next, 3.0 × 10^3^ sporozoites from each treatment were added to wells with HC-04 cells in triplicates. The plate was centrifuged at 200×*g* for 5 min, and sporozoites were allowed to invade HC-04 cells for 1 h at 37 °C in CO_2_ incubator. After one hour, the plates were washed with media to remove uninvaded sporozoites and a subsequent media change at 24 hpi. At 48 hpi, infected HC-04 wells were incubated with 1 × Xenolight (PerkinElmer) for 10 min at 37 °C. The plates were imaged on the CLARIOstar^*Plus*^ (BMG LABTECH) immediately after Xenolight incubation for relative luminescence units (RLU) output.

### RNA isolation, cDNA synthesis, and qRT-PCR

RNA was isolated from Pb-PfCSP and PbmCh-luc infected liver samples for qRT-PCR. Infected liver samples were homogenized in TRIzol (Fisher Scientific) on a TissueLyser II (Qiagen). RNA was coprecipitated with glycogen (Sigma-Aldrich) at − 20 °C overnight and treated with DNase (Fisher Scientific). cDNA was synthesized using SuperScript III Platinum two-step quantitative reverse transcription-PCR (qRT-PCR) kit (Invitrogen), according to manufacturer’s instructions. The LS burden was quantified by absolute copy number of *P. berghei* 18S rRNA (PBANKA_0521221) normalized to murine GAPDH (glyceraldehyde-3-phosphate dehydrogenase) (NM_001289726.2) copy number as previously described [57].

Sporozoite surface protein essential for liver stage development (SPELD, PBANKA_0910900) and liver-specific protein 2 (LISP2, (PBANKA_1003000) served as early and late LS markers, respectively (Additional file 1: Figure S1) [58]. Primer sequences for all genes are listed in Additional file 1: Table S1 [59]. PCR-amplified target cDNA inserted into pJET1.2 plasmid (Thermo Fisher Scientific) and standards were prepared at tenfold dilutions from 10^8^ through 10^1^. cDNA samples were quantified by Luna® Universal qPCR Master Mix (New England BioLabs) along with the standards and copy numbers were used to determine the gene expression.

### Statistical analysis

Significance between means was determined by a two-way ANOVA followed by a Tukey’s multiple comparisons post hoc test with relevant comparisons for sporozoite gliding assay. Where indicated, a Kruskal–Wallis test with Dunn’s multiple comparisons and a two-tailed Mann–Whitney test were used for analysing non-parametric data. IC_50_ curves were calculated using nonlinear regression dose–response modelling with concentrations converted to log_10_ in ILSDA. Correlation coefficients were determined using a two-tailed Spearman’s correlation. All graphs presented are displayed as means with standard error of the mean (SEM.), and data were analysed using GraphPad Prism version 9 (GraphPad Software, San Diego, California, USA). % Inhibition was calculated by the equation below:

$$\% Inhibition=100-\left[\frac{x}{Average\, of\, control}\times 100\right]$$

where the antibody-mediated inhibition being measured is represented as ‘x’.

## Results

### Monoclonal antibodies against CSP block the live gliding of transgenic sporozoites

**Fig. 1:**
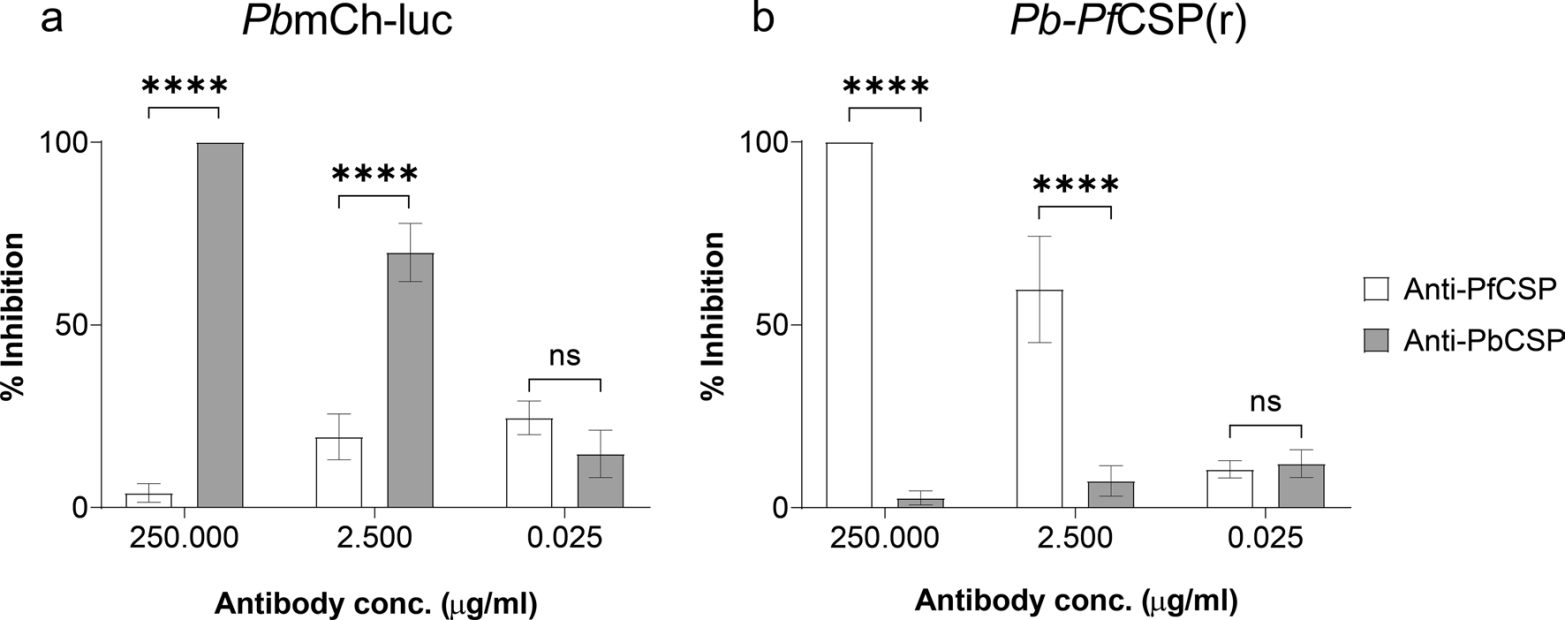
Anti-CSP monoclonal antibodies (mAbs) inhibit transgenic *P. berghei* gliding. **a** *Pb*mCh-luc treated with 3D11 (Anti-*Pb*CSP mAbs) and **b** *Pb*-*Pf*CSP(r) treated with 2A10 (Anti-*Pf*CSP mAbs) normalized to negative control. Column charts represent means ± SEM of triplicate wells in two biological replicates. A two-way ANOVA with Tukey’s multiple comparisons post-hoc test was used to determine statistical significance for all means and is presented as *P* < *0.0001 *(****) or *ns* not significant where indicated

To determine the range of mAb concentrations that achieved 100% to no inhibition in vitro, functional inhibition against transgenic *P. berghei* sporozoites in a live gliding assay was evaluated first (Fig. 1). The antibodies 3D11 (anti-*Pb*CSP) and 2D10 (anti-*Pf*CSP) inhibited sporozoite gliding in a dose-dependent manner and were not cross-reactive as *Pb*mCh-luc exposed to 2A10 and *Pb*-*Pf*CSP(r) exposed to 3D11 exhibited gliding motility similar to control (Fig. 1a and b). At a concentration of 2.5 µg/ml, mAbs 3D11 and 2A10 showed 69% and 59% inhibition to *Pb*mCh-luc and *Pb*-*Pf*CSP(r) sporozoites respectively. Complete inhibition of *Pb*mCh-luc and *Pb*-*Pf*CSP(r) live sporozoite gliding was observed at a concentration of 250 µg/ml for mAbs 3D11 and 2A10, respectively (Fig. 1a and b).

### ***Determination of the half-maximal inhibitory concentration (IC***_***50***_***) of anti-CSP mAbs by ILSDA***

**Fig. 2:**
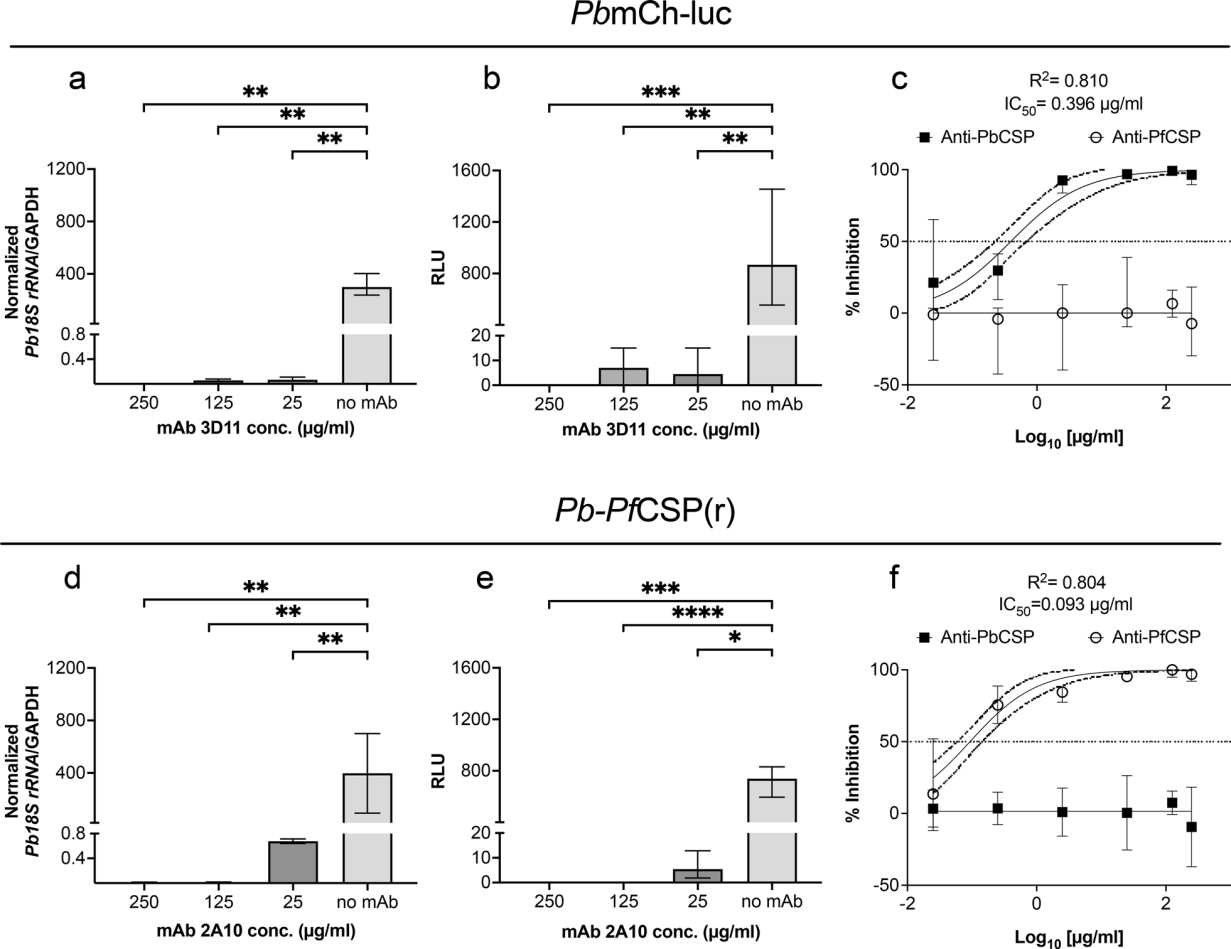
In vitro liver-stage burden quantification and IC_50_ determination of monoclonal antibodies. Quantification of *P. berghei* infection burden at 48 hpi under the most inhibitory concentrations (> 90%) in vitro was assessed by **a**, **d** qRT-PCR and **b**, **e** RLU for *Pb*mCh-luc and *Pb*-*Pf*CSP(r). Anti-CSP mAbs inhibition of liver-stage development of **c** *Pb*mCh-luc and **f** *Pb*-*Pf*CSP(r) was calculated using RLU and normalized to the control. IC_50_ curves were computed using Nonlinear regression dose–response modelling in Prism (GraphPad, La Jolla, CA, USA). A Kruskal–Wallis test with Dunn’s multiple comparisons determined statistical significance and is represented as *P* < *0.05 *(*), *P* < *0.005* (**), *P* < *0.0005* (***), *and P* < *0.0001* (****). All results are denoted as median ± 95% CI of triplicate wells in two biological replicates

Then, an ILSDA was performed to determine the IC_50_ of anti-CSP mAbs and assess the inhibition of LS forms. Luminescence of all concentrations tested and qRT-PCR for the highest inhibitory concentrations quantified parasite burden (Fig. 2a–e). Through relative luminescence, the IC_50_ values were determined to be 0.396 µg/ml and 0.093 µg/ml for 3D11 and 2A10, respectively (Fig. 2c and f). Therefore, 2A10 appeared to have approximately fourfold higher inhibition against *Pb*-*Pf*CSP(r) than 3D11 against *Pb*mCh-luc (Fig. 2c and f). High levels of inhibition (> 93%) by 3D11 at 250, 125, and 25 µg/ml were observed in *Pb*mCh-luc LS forms. At comparable concentrations of 2A10, near complete inhibition (> 92%) was observed of *Pb*-*Pf*CSP(r) LS forms (Fig. 2).

### Decreased transgenic parasite liver burden in mice that received anti-CSP mAbs

**Fig. 3:**
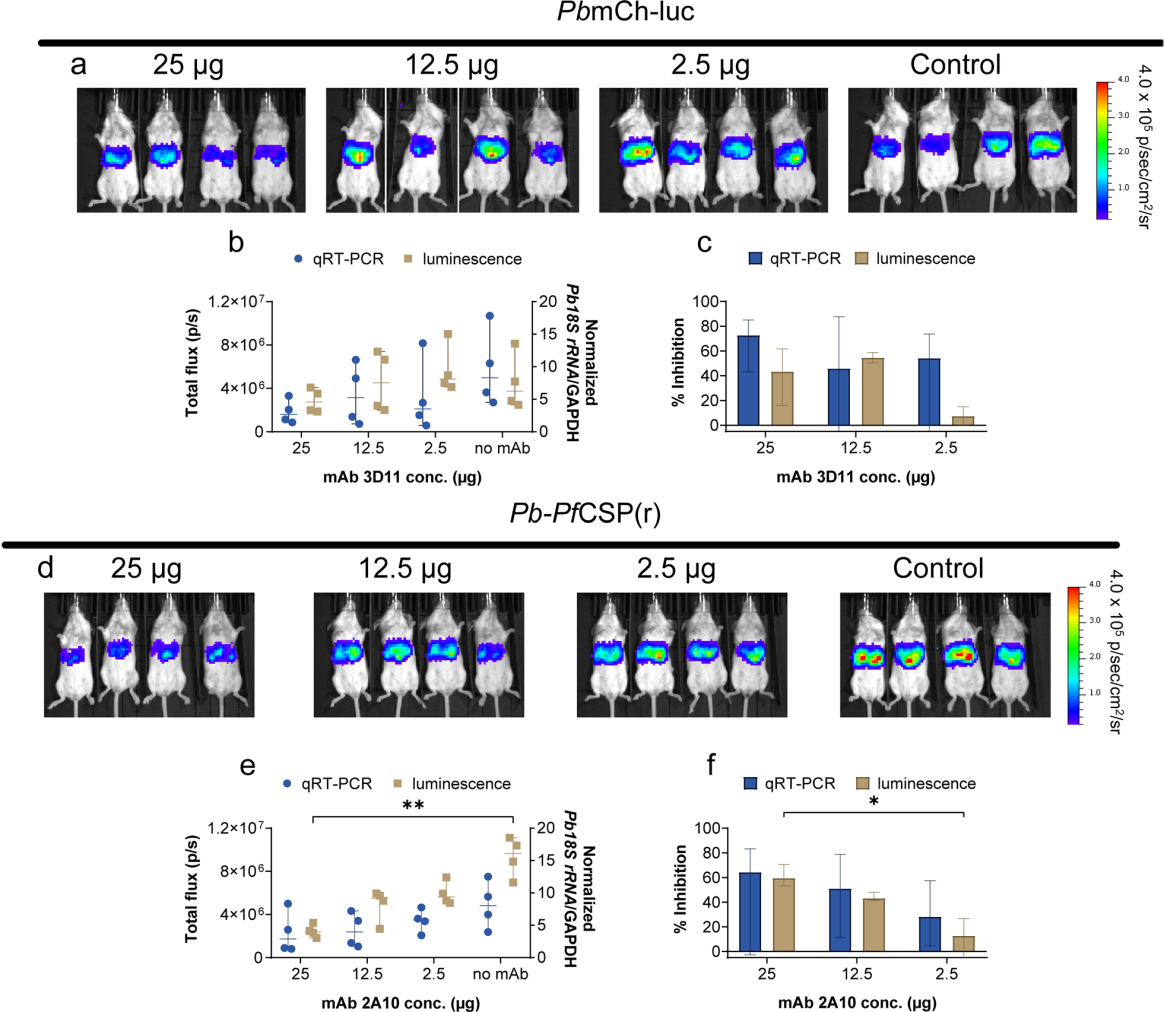
Passive transfer of anti-CSP mAbs reduce liver stage burden of transgenic *P. berghei* in mice. BALB/c mice were injected with 25 µg (250 µg/ml), 12.5 µg (125 µg/ml), and 2.5 µg (25 µg/ml) of anti-CSP mAbs (n = 4 per group) 30 min before the challenge with 5 × 10^3^ sporozoites via tail vein injection. Representative images of **a** *Pb*mCh-luc and **d** *Pb*-*Pf*CSP(r)-infected mice quantified at 44 hpi by **b**, **e** qRT-PCR and bioluminescent imaging. No antibody-treated mice (n = 4) were used to normalize data for **c**, **f** percent inhibition. Results are represented as median ± 95% CI for each experimental group. Statistical significance was calculated using a Kruskal–Wallis test with Dunn’s multiple comparisons and defined as *P* < *0.05* (*) and *P* = 0.0099 (**)

To assess the functional inhibition in vivo, mice that were given the mAbs at the highest inhibitory concentrations (250, 125, and 25 µg/ml) received sporozoite challenge infections. Upon challenge with transgenic *P. berghei*, IVIS imaging was done at 44 h as shown (Fig. 3a and d). Total flux (p/s) and qRT-PCR quantified liver burden (Fig. 3b and e) while control mice were used to normalize data and calculate percent inhibition (Fig. 3c and f). 2A10 significantly inhibited liver-stage development at 25 µg compared to 2.5 µg (p = 0.0148) in *Pb*-*Pf*CSP(r) infected mice (Fig. 3f). However, no significant reduction in liver burden was observed in mice that received 3D11 (Fig. 3c).

### Correlation of functional inhibition of anti-CSP mAbs between in vitro and in vivo assays

**Fig. 4:**
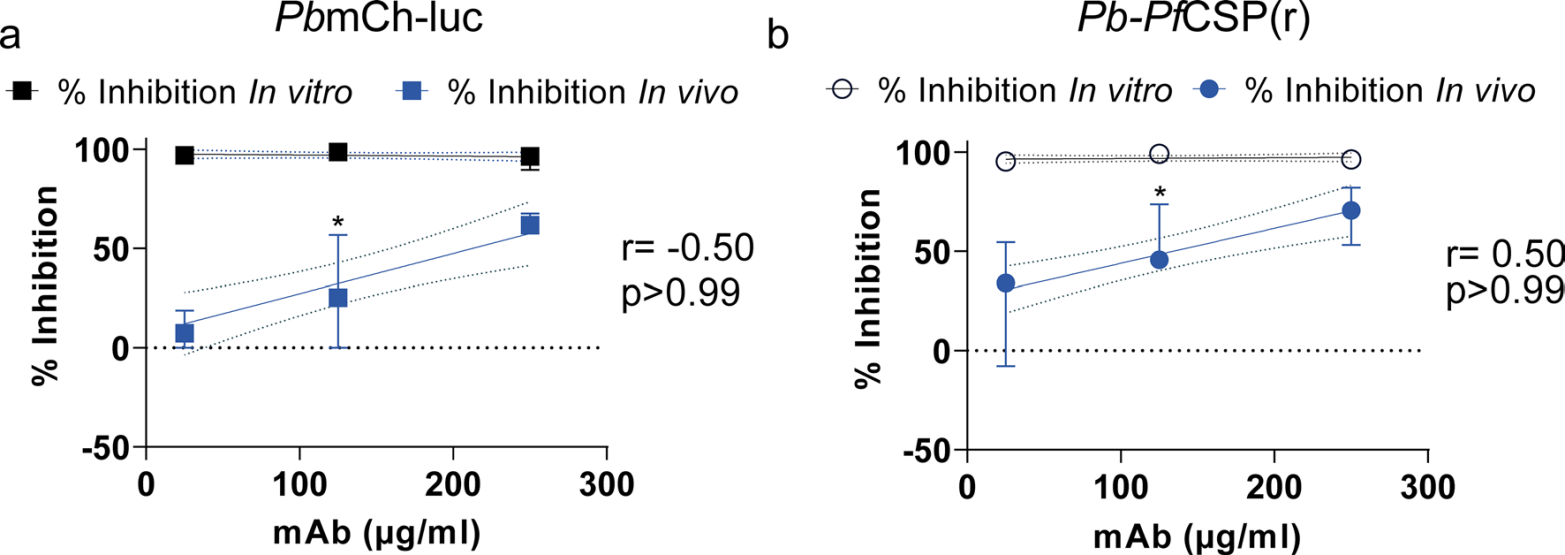
Correlation between in vitro and in vivo inhibition of anti-CSP monoclonal antibodies. Inhibition percentage normalized to control for relative luminescence between in vitro and in vivo assays of **a** *Pb*mCh-luc and **b** *Pb*-*Pf*CSP(r) at 250, 125, and 25 µg/ml concentrations of anti-CSP 3D11 and 2A10 respectively. Correlation was tested using Spearman’s r test in Prism (GraphPad, La Jolla, CA, USA) with r and p-values in text. ‘*’ indicates this in vivo dilution represents 4 technical replicates. Results are represented as median ± 95% CI

Lastly, since a dose-dependent antibody reduction of both parasite lines were observed at equivalent concentrations in vivo, the relationship between functional inhibition in vitro to in vivo was explored further (Fig. 4). Additionally, as relative luminescence possessed a good correlation with qRT-PCR of infected mice liver samples (Additional file 1: Figure S2), the focus was narrowed to comparing inhibition by bioluminescence across both assays. Although the highest concentrations (250, 125, and 25 µg/ml) tested in vitro achieved 92–100% inhibition of liver-stage invasion, in vivo the efficacy of these concentrations ranged widely from ~ 0–60% for 3D11 and ~ 30–68% for 2A10. Hence, no significant correlation was observed between the two assays at overlapping concentrations determined by luminescence (Fig. 4a and b).

## Discussion

Functional assays replicate critical invasive sporozoite phenotypes in vitro [32, 34–38, 46, 55, 60]. These bio-assays are paramount in initially quantifying functional antibody-mediated inhibition. Additionally, rodent malaria challenge models are used to characterize humoral immune responses [39, 61]. Thus, it is necessary to elucidate how antibody-mediated inhibition in vitro translates into inhibition in vivo. To accomplish this, the study utilized two well-characterized transgenic lines (*Pb*mCh-luc [53] and *Pb-Pf*CSP(r) [52, 62]) and inhibitory anti-CSP monoclonal antibodies. The inhibition potential and concentration of mAbs 2A10 and 3D11 were validated by comparative analysis in a live sporozoite gliding assay (Fig. 1). Monoclonal antibodies were not cross-reactive as the B-cell epitopes of 3D11 and 2A10 are the immunodominant central repeat region of *P. berghei* and *P. falciparum* CSP, respectively [48, 49, 63, 64]. Hence, 2A10 mAbs bind strongly,K_D_ of 2.7 ± 2.1 nM [65], to ‘NANP’ repeats in *P. falciparum* CSP while 3D11 mAbs have a high affinity,K_D_ of 159 ± 47 nM [64], for repeats ‘PAPP’, ‘NAND’, and ‘NPND’ in *P. berghei* CSP [63, 64].

Next, IC_50_ concentrations were determined against in vitro liver-stage development using HC-04 cells. The observed disparity between IC_50_ values of 2A10 and 3D11, despite antibodies exhibiting comparable inhibition at the highest concentrations tested, may be related to differences in epitope binding affinity and avidity which was not explored in this study (Fig. 2). The IC_50_ value for 2A10 against transgenic *Pb*-*Pf*CSP(r) (Fig. 2) was similar to previously published data for 2A10 against *P. falciparum* LS forms in primary human hepatocytes [32]. However, inhibition varied marginally in Rodríguez-Galán et al., using 2A10 mAbs and *Pb*-*Pf*CSP(r), with an observed IC_50_ of 0.4 µg/ml [51]. This discrepancy was only observed for 2A10. The functional inhibition for 3D11 was shown to be effective at 1 µg/ml against both *Pf*CSP@*Pb*UIS4 [51] and *Pb*mCh-luc (Fig. 2). Similar inhibitory concentrations for 3D11 were reported using a *P. berghei* sporozoite cell traversal assay [66]. Therefore, these results corroborate previously published studies where both 2A10 and 3D11 had similar functional inhibition in vitro.

Following determination of the IC_50_ in vitro, an in vivo challenge was conducted. The mouse study was restricted to the highest inhibitory concentrations (> 90%) as the lower concentrations assessed in vitro had less than 80% inhibitory. Previous studies required a higher dose of 2A10 to reduce liver burden compared to the in vivo data from this study [45, 63, 67]. Transgenic *P. berghei* liver burden was reduced by 75% to 82% in C57BL/6 mice that received 300 µg of 2A10 [63]. However, in humanized FRG HuHep mice, 87% to 99% inhibition was achieved after a passive transfer of 500 µg of 2A10 mAbs and challenged with 2 × 10^6^ *P. falciparum* sporozoites [45]. In a mosquito-bite challenge, 51% inhibition of liver-stage burden was observed after 24 h of mice being passively given 150 µg of 2A10 [67]. Despite a similar reduction in liver burden, reduced sporozoite speed, and blood vessel invasion in mice passively immunized with 150 µg and 25 µg of 3D11 [68], a significant reduction in liver burden was not observed for 3D11 at the concentrations tested.

In the current study, the difference between 3D11 inhibition of *Pb*mCh-luc in vitro and in vivo was lower than between 2A10 inhibition of *Pb*-*Pf*CSP(r) in vitro and in vivo (Fig. 4). These findings suggest the potential for alternative antibody-mediated mechanisms of inhibition in vivo versus in vitro. Previously, in a cell traversal assay, a group of antibodies including 2A10 demonstrated differential inhibition in vivo that was not observed in vitro [67]. High affinity and avidity of mAbs determined in vitro were better predictors of protective efficacy in vivo [46, 69]. This study provides valuable insight into the predictably of in vitro bioassays to in vivo model outcomes; however, some limitations include the fact that antibody concentration after IP injections was not explored thereby limiting potential correlation to the dosage of mAbs administered. Secondly, the sample size was not enough to make definitive conclusions and lastly, mice were challenged intravenously, restricting inhibitory mAb activity to a short timeframe of sporozoite inoculum in blood. Route of sporozoite administration plays an essential role as antibodies effectively inhibit sporozoites at the site of injection [61, 68, 70, 71]. Future studies will explore alternative routes of infection including mosquito bites, intradermal or subcutaneous injections, replicating other humoral mechanisms in vitro with a larger cohort of mice*,* and comparing inhibition in vitro across primary cells, immortal cell lines and other rodent models [72, 73]. This comprehensive approach would give a better understanding of factors influencing the translatability of antibody-mediated inhibition in vitro to in vivo outcomes.

## Conclusions

This study evaluated antibody-mediated inhibition of transgenic *P. berghei* LS forms in in vitro and in vivo assays. While 2A10 mAbs had higher inhibitory activity in vitro and in vivo than 3D11 mAbs, the discrepancy in inhibition between both assays was more pronounced for 2A10 than 3D11. Despite some similarities between antibody inhibition titration curves, no significant correlation was established in parasite burden between these assays by luminescence. While not optimal for predicting in vivo outcomes, in vitro assays remain a useful preliminary screening tool as high throughput cost-effective laboratory assays for vaccine discovery.

### Supplementary Information

**Additional file 1: Figure S1.** Gene expression levels of early and late liver-stage *P. berghei* biomarkers. **Figure S2.** Correlation between relative luminescence and *P. berghei* 18S rRNA expression in infected mice. **Table S1.** Primers used in qRT-PCR for LS quantification and maturity biomarkers.

## Data Availability

The data analysed from this study are available from the corresponding author upon reasonable request. All relevant data are present in the manuscript.

## Abbreviations

NIAID: National Institute of Allergy and Infectious Disease
NIH: National Institutes of Health
BSA: Bovine serum albumin
GAPDH: Glyceraldehyde-3-phosphate dehydrogenase
mAbs: Monoclonal antibodies
ILSDA: Inhibition of liver-stage development assay
LS: Liver stage
