# Supplementary material for: Comparative analyses of functional antibody-mediated inhibition with anti-circumsporozoite monoclonal antibodies against transgenic Plasmodium berghei
Source: Malar J. 2023 Nov 7;22:335. doi: 10.1186/s12936-023-04765-2 (PMC10629016; doi:10.1186/s12936-023-04765-2)
Supplement: Supplementary file 1 — Additional file 1: Figure S1. Gene expression levels of early and late liver-stage P. berghei biomarkers. Figure S2. Correlation between relative luminescence and P. berghei 18S rRNA expression in infected mice. Table S1. Primers used in qRT-PCR for LS quantification and maturity biomarkers. [file 12936_2023_4765_MOESM1_ESM.docx]

***Additional materials***

**Figure S1: Gene expression levels of early and late liver-stage *P. berghei* biomarkers.**

Gene expression levels of sporozoite surface protein essential for liver stage development (SPELD) and liver-specific protein 2 (LISP2) normalized to host GAPDH were quantified by qRT-PCR for (a) *Pb*mCh-luc and (b) *Pb*-*Pf*CSP(r) *in vitro* and *in vivo*. Statistical significance was determined by a two-tailed Mann-Whitney test and represented *as P<0.005 (**), P=0.0006 (***), and P<0.0001 (****).*

**Figure S2**: **Correlation between relative luminescence and *P. berghei* 18S rRNA expression in infected mice.** Spearman’s r correlation for (n=4) for (a) *Pb*mCh-luc for qRT-PCR and luminescence was 0.2, while (b) qRT-PCR and luminescence correlation of *Pb*-*Pf*CSP(r) was 1.0.

**Table S1:** Primers used in qRT-PCR for LS quantification and maturity biomarkers**.**

| Primer name | Primer Sequence (5’ to 3’) |
| --- | --- |
| *Pb*18S rRNA FWD | GGGAGATTGGTTTTGACGTTTATG |
| *Pb*18S rRNA REV | TAAGGATGTATTCGCTTTATTTAATGCTT |
| Mouse GAPDH FWD | GACCTCAACTACATGGTCTACATG |
| Mouse GAPDH REV | CATCACCATCTTCCAGGAGC |
| SPELD FWD | TATTTATTACCCTGCGGATA |
| SPELD REV | ATACTCAACGTGATATTTCCA |
| LISP2 FWD | GCATTACTACTGAATCTTCATCGTGCACATT |
| LISP2 REV | GTATTTCCATTTGCGTCAATATCCTCAAG |
